# Immunohistochemistry-Based Molecular Profiling of Muscle-Invasive Bladder Cancer: Analysis of 100 Consecutive Cases with Morphological Correlation

**DOI:** 10.3390/medsci13030202

**Published:** 2025-09-22

**Authors:** Elitsa Kraevska, Savelina Popovska

**Affiliations:** Department of Pathoanatomy, Faculty of Medicine, Medical University-Pleven, 5800 Pleven, Bulgaria

**Keywords:** muscle-invasive bladder carcinoma, molecular variants, immunohistochemistry, histological subtypes

## Abstract

**Background/Objectives**: This study aimed to profile the molecular variants of muscle-invasive bladder cancer (MIBC) based on immunohistochemical analysis and to make a correlation with morphological characteristics in a series of 100 consecutive patients. **Methods**: A retrospective single-center study was conducted on 100 consecutive cases of MIBC (2021–2024). A selected immunohistochemical (IHC) panel (including CK5/6, CK20, and p16) was applied in all cases to classify the tumors into known molecular variants (luminal papillary, luminal non-specified, luminal unstable, stroma-rich, basal/squamous, neuroendocrine-like). **Results**: Seven molecular subtypes are identified: basal (33%), luminal papillary (24%), luminal unstable (16%), luminal non-specified (10%), basoluminal (double-positive) (9%), neuroendocrine-like (double-negative with neuroendocrine morphology) (6%), and stroma-rich (2%). This distribution largely matches published data (Consensus Classification and The Cancer Genome Atlas (TCGA)), with minor differences (e.g., a lower share of the stroma-rich variant). A strong correlation is found between the histological subtypes of some tumors and their molecular variant (χ^2^, *p* < 0.001): for example, all cases of urothelial carcinoma with squamous differentiation are basal, micropapillary tumors are entirely luminal, and small-cell carcinomas are neuroendocrine-like. **Conclusions**: The results demonstrate that the morphological subtype of urothelial carcinoma largely predetermines the molecular profile. Combining classic histopathology with IHC-based profiling allows for a more complete characterization of the tumor and aids prognosis and personalized treatment in MIBC.

## 1. Introduction

Bladder cancer is the 10th most common malignancy worldwide, accounting for about 3% of all newly diagnosed cancer cases [1]. The disease predominantly affects men (approximately four times more frequently than women) and usually arises in older age—over 90% of patients are above 55 years, with a typical median age at diagnosis of ~70 years [1]. Epidemiological data also show sex differences in its course: although men develop bladder cancer more often, women are frequently diagnosed at a more advanced stage and higher grade, leading to a worse prognosis [2]. Differences in survival disadvantaging women are observed especially in the first 1–2 years after diagnosis, which is attributed both to possible delays in diagnosis (e.g., misattributing hematuria in women to cystitis) and to biological sex differences [2]. It is thought that immunological, hormonal, and genetic factors contribute to the more aggressive course in women [2]. Despite these differences, therapeutic guidelines (EAU, NCCN) do not stratify treatment by sex; management strategy is determined primarily by tumor stage and grade.

Histologically, over 90% of bladder tumors are urothelial carcinomas arising from the urothelial lining [1]. The remaining <10% consist of other rare histological types—most commonly squamous cell carcinoma (~5%, associated with chronic irritation or schistosomiasis) and adenocarcinoma (including those with urachal origin) [1]. The World Health Organization classifies urothelial tumors by grade—low-grade vs. high-grade—and also separates carcinoma in situ (CIS) as a flat high-grade lesion. This classic pathological classification is prognostically important, as non-invasive tumors (pTa, pT1) have excellent outcomes with timely therapy, whereas invasive tumors (invasion into the muscle layer—pT2 or higher) are associated with significantly lower survival [1,3]. Approximately 25–30% of urothelial carcinoma cases are initially muscle-invasive (stage ≥ T2), for which the standard treatment is radical cystectomy with or without neoadjuvant/adjuvant therapy [1].

Over the past decade, intensive molecular-genetic studies have led to the development of a molecular classification of MIBC. Global transcriptomic profiles reveal two major molecular phenotypes—luminal and basal, analogous to those in breast carcinoma [3]. Luminal tumors are characterized by a gene expression pattern resembling that of differentiated urothelium, whereas basal tumors have a profile similar to urothelial basal cells, often with features of squamous differentiation [3]. With accumulating data, “double-negative” variants have also been described in which expression of typical luminal or basal markers is lacking—some of these are characterized by abundant stroma or neuroendocrine morphology. In 2020, an international consensus classified muscle-invasive urothelial carcinoma into six molecular classes: luminal papillary (LumP), luminal non-specified (LumNS), luminal unstable (LumU), basal/squamous, stroma-rich, and neuroendocrine-like [4]. Some systems additionally describe a mixed “basoluminal” phenotype with co-expression of features of both main classes [3,5].

The molecular variants differ in their biology and clinical behavior. Knowledge of the frequency and characteristics of each variant is important, as some have distinct prognoses and therapy sensitivities. For example, basal and stroma-rich cancers typically show a better response to platinum-based chemotherapy, whereas luminal papillary cancers are associated with a more favorable prognosis but poor response to systemic chemotherapy (in such cases, radical surgery and/or targeted therapy—e.g., FGFR inhibitors if FGFR3 mutations are present—are more appropriate) [6]. Clinically, immunohistochemistry remains an indispensable tool in the diagnosis and prognostication of bladder cancer, and it can be used as a more accessible method for molecular profiling and for validating transcriptomic classifications in routine practice. Ongoing harmonization of classifications and their confirmation in a clinical context (including via standard IHC panels) will facilitate implementation of molecular subtyping to personalize treatment for MIBC patients. The present study aims to perform immunohistochemical and morphological profiling of the molecular variants in muscle-invasive urothelial carcinoma. We present the results from a series of 100 consecutive MIBC cases, comparing the frequency of the identified molecular variants with published data in the literature (consensus classification [4] and the TCGA classification [7]. We examine the similarities and differences in variant distribution, as well as the interrelationships between the histological subtypes of the tumors and their molecular profile.

## 2. Materials and Methods

### 2.1. Clinical Cohort and Study Design

A retrospective single-center study was carried out on patients with MIBC treated at the Urology Clinic of University Hospital “St. Marina”—Pleven, Bulgaria over a 4-year period (January 2021–December 2024). The study series includes 100 consecutive patient cases with histologically confirmed muscle-invasive urothelial carcinoma of the bladder who underwent either radical cystectomy (RC) or transurethral resection (TURB) of the tumor. The ratio of surgical intervention types is 34% RC to 66% TURB. Tumors were staged and graded according to the fifth edition of the World Healt Organization (WHO) classification of urogenital tumours published in 2022 [8].

**Figure 1:**
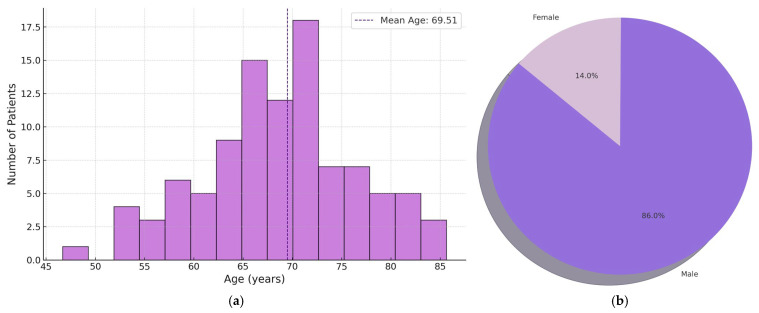
Distribution of MIBC patients (n-100): (**a**) by age; (**b**) by gender.

Inclusion criteria: primary muscle-invasive urothelial carcinoma of the bladder (≥pT2 at diagnosis). Exclusion criteria: non-muscle-invasive tumors (pTa, pT1), recurrence in the same patient, and insufficient tumor material (exhausted biopsies). For all included cases, data were collected from hospital records: age, sex, stage, and histological subtype of the carcinoma. The key demographic indicators of the patients are as follows: mean age 69.5 years (standard deviation 8.7; median 70; range 47–87 years) and male sex in 86% of cases (Figure 1). Males greatly predominated over females (86% vs. 14%), which corresponds to the known epidemiology of the disease. Over 90% of patients were over 55 years old, and the median, ~70 years, also matches expectations. Of all 100 tumors, 73% had pure morphology (presence of only one subtype classified by WHO), while 27% exhibited mixed histology—i.e., the presence of more histological subtypes within the same neoplasm.

### 2.2. Immunohistochemical Analysis and Molecular Subtyping

All cases were evaluated independently by two pathologists (one professor (SLP) and one uropathologist (EPK)), and paraffin blocks with invasive tumor involving the muscularis propria were selected for immunohistochemical analysis. Four-micron sections were cut onto adhesive slides and processed by a standard protocol for IHC technique on an automated platform (Dako/Agilent). A panel of antibodies was used, selected as a surrogate marker panel to distinguish the major molecular variants. The following primary antibodies were included: CK5/6 (mouse, monoclonal, clone D5/16 B4, ready to use, Dako/Agilent, targeting cytokeratins 5 and 6), CK20 (mouse, monoclonal, clone Ks20.8, ready to use, Dako/Agilent targeting 20 kDa cytokeratin), and p16 (mouse, monoclonal, clone JC2, ready to use, Diagnostic BioSystems, California, U.S. targeting the tumor suppressor protein). Antigen retrieval was performed by heat-induced epitope retrieval (HIER)—optimally in an alkaline buffer (e.g., Tris-EDTA, pH 9) for ~20 min at ~95 °C. Primary antibody incubation was 20–30 min at room temperature, followed by visualization with a two-step polymer HRP/DAB system (EnVision) to develop the signal. Appropriate positive controls were used for each marker: normal prostatic glands for CK5/6 (strong positive staining in basal cells), appendix for CK20 (diffuse cytoplasmic staining of the surface epithelium and moderate in crypts), and high-risk HPV-associated squamous cell carcinoma of the uterine cervix for p16 (diffuse strong nuclear and cytoplasmic expression).

**Figure 2:**
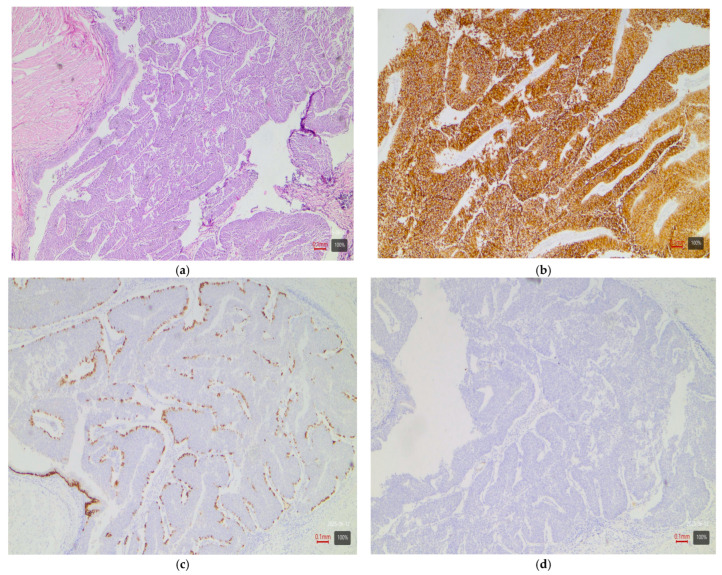
UC with papillary architecture—LumP variant: (**a**) H&E; (**b**) CK20-positive (+ve); (**c**) CK 5/6-negative (−ve) staining of a basal membrane-like pattern; (**d**) p16-negative (−ve).

The IHC staining is evaluated semi-quantitatively by the pathologists. Although the H-score method (combining percentage and intensity of staining) is widely used, its application is challenging in cases of heterogeneous expression. In the present study, we adopted a simpler positivity criterion—the presence of specific tumor staining in over 10% of the tumor cells. For each tumor, CK5/6 and CK20 are assessed by intensity (strong, moderate, weak) and percentage of positive cells (in 10% increments). CK5/6 is used as a surrogate marker for a basal molecular phenotype and CK20 as a marker for a luminal phenotype. A tumor is considered CK5/6-positive (hence “basal”) if there is strong or moderate cytoplasmic staining in ≥10% of the cells (staining of a basal membrane-like pattern is interpreted as a negative result (Figure 2c). Similarly, CK20-positive tumors (≥10% of cells with strong or moderate cytoplasmic staining) are considered luminal.

For further differentiation of the luminal tumors, we performed p16 immunostaining. We took into account literature data suggesting that normal urothelium often does not express or only weakly expresses p16, whereas overexpression of p16 in tumors may indicate genomic instability. In our study, we interpret p16 as positive if there is intense or moderate nuclear staining in ≥50% of tumor cells or so-called “block” positivity (staining of ≥50% of both the nucleus and cytoplasm in tumor cells), and weak or absent nuclear staining (regardless of cytoplasmic staining) is considered a negative result.

Based on the combination of marker expression, each tumor is classified into a corresponding molecular variant as follows:

1. Basal variant: CK5/6 (+ve) and CK20 (−ve).
2. Luminal variant: CK20 (+ve) and CK5/6 (−ve). The luminal tumors are further subdivided: luminal papillary if p16 is negative, and luminal unstable if p16 is positive. Luminal non-specified variant: preserved urothelial morphology, expression of either marker (CK5/6 (−ve), CK20 variable (−ve or <10%)). These cases phenotypically fall into the luminal spectrum but do not classify as luminal papillary or luminal unstable.
3. Double-negative variants: CK5/6 (−ve) and CK20 (−ve). These tumors are split into two subgroups according to morphology: cases with neuroendocrine morphological characteristics are classified as neuroendocrine-like variant and cases with abundant stroma and/or sarcomatoid areas as stroma-rich variant.
4. Basoluminal variant: tumors co-expressing both CK5/6 and CK20 (co-expression). In our series, such cases had mixed morphology, with different parts of the tumor showing zonal expression of different markers (e.g., one tumor component basal, another luminal).

### 2.3. Statistical Analysis

Data processing was performed using SPSS v.25 software (Statistical Package for the Social Sciences v.25 (SPSS Inc., Chicago, IL, USA)). Descriptive statistics (mean values, proportions) were used to characterize subtype distribution. The relationship between categorical variables (e.g., histological subtype and molecular variant) was examined with a chi-square test for independence. A significance level of *p* ≤ 0.05 was considered statistically significant.

### 2.4. Ethical Aspects

This study was conducted following the national and international requirements for clinical studies, including the preservation of the anonymity of the participants and the non-disclosure of their personal information. Each participant signed an informed consent form. This study was conducted in accordance with the requirements of the Ethics Committee of the Medical University of Pleven, Bulgaria, approval number No 781/14.06.2024.

## 3. Results

### 3.1. Pathological Characteristics of the Patients

The most common subtypes are urothelial papillary carcinoma (27 cases, 27%) and urothelial carcinoma with squamous differentiation (present as a component in 17 tumors, 17%), followed by less common subtypes such as sarcomatoid urothelial carcinoma (5 cases, 5%), small-cell carcinoma (5 cases, 5%), micropapillary urothelial carcinoma (4 cases, 4%), urothelial carcinoma with glandular differentiation (3 cases, 3%), plasmacytoid urothelial carcinoma (3 cases, 3%), poorly differentiated urothelial carcinoma (3 cases, 3%), and nested urothelial carcinoma (2 cases, 2%) (Table 1). Some tumors (27% in total) have mixed morphology—for example, a conventional urothelial carcinoma combined with other histological subtype components in varying proportions. In the mixed group, the tumors were distributed as follows: 16 cases of conventional UC + clear-cell histological characteristics, 1 case of lymphoepithelioma-like + giant-cell subtype, 2 cases of conventional UC + microcystic subtype, 1 case of lipid-rich + UC with glandular differentiation, 1 case of conventional UC + sarcomatoid subtype, 1 case with glandular differentiation + conventional UC + clear-cell subtype, 1 case of conventional UC + plasmacytoid, 1 case of UC with squamous differentiation + clear-cell subtype + conventional UC, 1 nested + conventional UC, 1 plasmacytoid + clear-cell subtype + UC with squamous differentiation, and 1 case of conventional UC + UC with glandular differentiation + small-cell carcinoma. Our study on this heterogeneous group is ongoing as it represents a therapeutic challenge. Whether to treat the more aggressive histological subtype or the one represented in a higher percentage in the biopsy sample is still unknown.

We found that 66% (including mixed tumors) of MIBC cases have at least one divergent histological component. This is higher than the classically cited 20–30% incidence of variant differentiation in bladder cancer [6]. One reason may be the meticulous search for and reporting of even minimal foci of variant differentiation in our study. It is known that in routine practice, up to 50% of variant components can initially remain unrecognized, especially if they are small foci. In our series, purposeful, thorough examination of all resection specimens likely led to more complete identification of these components. Another factor is case selection: all cases we studied are muscle-invasive (pT2+), whereas statistics from the literature often include non–muscle-invasive (superficial) tumors, and invasive tumors have a higher tendency toward divergent differentiation. Our data may also reflect that more complex, “atypical” cases are referred to a university hospital, thereby increasing the proportion of rare variants in the cohort.

The specific frequencies of variants in our study largely fall within known ranges. Squamous differentiation (17% of cases) is in line with expectations—it is the most common divergent form in urothelial carcinoma, with a reported incidence up to 40% [9]. Some authors even note that if one deliberately searches for even minimal squamous foci, the proportion can reach 60%, which highlights how widespread (though often underappreciated) squamous metaplasia is in urothelial tumors. Glandular differentiation (3% in our series) is slightly below the typical 10–20% [10]; it is possible that some of our mixed cases with minor glandular areas were categorized simply as “mixed variant” without separately noting the glandular component. The micropapillary variant (4% of cases) falls within the usually cited range of 2–5% [9]. The plasmacytoid variant (3%) is above literature frequencies of 1% [9,10]—it is rare but very aggressive, often being diagnosed at an advanced stage (pT3/T4) and with a high rate of peritoneal carcinomatosis. The nested variant (2%) is above expectations (less than 1% [9])—this likely reflects an increased vigilance for this otherwise hard-to-recognize variant, which is often mistaken for benign changes. The sarcomatoid variant (5%) and small-cell carcinoma (5%) are significantly above literature frequencies (<1% each [9,10]), but these deviations are probably an artifact of the small sample size and chance (5 cases out of 100). Nevertheless, the presence of these subtypes underscores the aggressiveness of the population studied.

In conclusion, regarding the morphological findings, our series supports the notion that tumors with variant histology are not an exception but rather the rule in muscle-invasive bladder cancer—over two-thirds of cases in our study had some divergent feature (squamous, glandular, sarcomatoid, etc.). This has direct clinical implications: it is advisable for pathologists to actively search for such variant features because identifying them can alter the tumor’s stage (for example, the presence of a sarcomatoid component automatically designates the tumor as high-grade/grade 3 and is often associated with more aggressive behavior) and inform therapeutic planning (for example, micropapillary tumors, even with limited invasion, often warrant early cystectomy due to their tendency for dissemination).

### 3.2. Molecular Classification of the Tumors

The high cost and limited accessibility of technologies for comprehensive molecular characterization restrict their use in routine clinical practice. Therefore, immunohistochemistry is employed as a more feasible surrogate to define the main molecular subtypes—luminal and basal—similar to the classification used in breast cancer. Choi et al. demonstrated that only two markers are sufficient to distinguish luminal (CK20+, CK5/6–) from basal (CK5/6+, CK20–) variants in bladder cancer [6]. Rebola et al. applied a similar approach and proposed a molecular grading system for non-muscle invasive bladder cancer, also identifying two additional groups: “double-positive” and “double-negative” [6]. Rodriguez-Pena et al. expanded the marker panel (GATA3, CK20, ER, Uroplakin II, and HER2 for luminal; CK5/6 and CD44 for basal phenotype) and confirmed that CK5/6 expression predicts stage progression in non-muscle invasive disease [6]. Han et al. reported correlations between molecular variants and pathologic features, showing that extensive squamous differentiation and sarcomatoid changes are associated with basal and claudin-low tumors, while the micropapillary variant can present with either luminal or basal phenotype [6]. Together, these findings support the use of immunohistochemistry as a practical method for basic assessment of luminal–basal variants in clinical practice, pending further validation studies.

Through IHC profiling, each tumor is classified into a particular surrogate molecular variant. In our series of 100 MIBCs, a total of 7 molecular variants are identified: basal—33% of cases; luminal papillary—24%; luminal unstable—16%; luminal non-specified—10%; basoluminal—9%; neuroendocrine-like—6%; stroma-rich—2%. The distribution of variants is summarized in Table 2, alongside a comparison with published data from large molecular studies. As can be seen, the frequencies of the major molecular categories in our study closely match those in the literature. The basal variant represents about one-third of all MIBC, both in our data (33%) and in the consensus classification (35%) and TCGA (35%). The luminal papillary variant is 24% in our cohort, identical to ~24% in the consensus, although in the TCGA series, this share is higher (~35%). The luminal unstable variant is also of similar frequency—~15–16% in both our series (16%) and the literature. The luminal non-specified variant constitutes a small portion (~6–10%) in all series. A noticeable discrepancy is observed for the stroma-rich variant: in the consensus classification, it accounts for ~15% of MIBC, whereas in our study, it is rare (only 2%). Conversely, the neuroendocrine-like variant (double-negative with neuroendocrine morphology) in our series is 6%, compared to ~3–5% in the literature (TCGA defines an analogous “neuronal” variant). The basoluminal variant is present in ~9% of our cases—similar “mixed” co-expression profiles have been noted by other authors (~5–10%), although they are not always delineated as a separate class.

In summary, the distribution of molecular variants in our series is similar to that reported in the major studies on molecular classifications of MIBC [4,7] Our data confirm the validity of the consensus taxonomy—the main variants are present with their expected proportions. The observed deviations (a higher or lower relative share of certain subtypes) likely reflect the limitations of the small sample and the specific characteristics of the studied population. A strong correlation is established between some histological subtypes of the tumor and their molecular variants. In brief, tumors with a given distinct differentiation show a preferred classification into a corresponding molecular class. This relationship is confirmed statistically (chi-square test, *p* < 0.001), and the most important patterns are described below.

## 4. Discussion

### 4.1. Comparison with Published Data on Molecular Variant

The obtained results from the IHC-based molecular classification confirm the main trends reported in the literature for MIBC. In our study, basal tumors constituted ~33%, which is almost identical to the ~35% relative share in the international consensus and in TCGA (Table 2). Thus, about one-third of all muscle-invasive carcinomas are consistently categorized as basal across different series. The basal (also known as basal/squamous) variant is characterized by high expression of basal cytokeratins (CK5/6, CK14) and low expression of the luminal markers CK20 and GATA3 [6]. It is often associated with squamous differentiation and tends to occur more frequently in women. Our data confirm the expected frequency of basal tumors and support the profile of this variant described in the literature.

The luminal papillary variant represented 24% of our cohort, aligning exactly with the 24% in the consensus. TCGA reported a higher percentage (~35%) of luminal papillary tumors, which is likely due to methodological differences—in the consensus classification, some cases that TCGA classified as luminal are reallocated into separate groups (e.g., “luminal unstable” and “luminal non-specified”). The luminal unstable (Luminal Unstable, LumU) variant in our study is 16%, virtually identical to the published ~15%. This variant corresponds to tumors with high mutational burden and an aggressive genomic profile—equivalent to the so-called “Genomically Unstable” class in some older classifications (Lund). TCGA did not define LumU as a separate category, but a subset of their luminal tumors with an infiltrated phenotype falls into this category. The luminal non-specified variant (Luminal Non-specified, LumNS) is represented in 10% of our cases, which is slightly above the ~6–8% reported in the large series. These are luminal tumors that do not fit into the papillary or unstable subtypes. TCGA identified a similar small group (~6%) of “pure luminal” tumors with a differentiated phenotype. In both our study and the large studies, this LumNS variant is the smallest luminal category.

More notable differences are observed for the stroma-rich (infiltrated) variant. Our share is only 2%, versus ~15–20% in the consensus classification and TCGA (Table 2). It is possible that stroma-rich tumors are less represented in our cohort or that some cases are classified by us as other subtypes (for example, some might overlap with the luminal-infiltrated category in TCGA). The neuroendocrine-like variant in ~6% of our patients is a slightly higher frequency than reported (3–5%). This variant encompasses tumors with neuroendocrine morphology. The differences here likely reflect random variation—our series contained five small-cell carcinomas and one poorly differentiated carcinoma with small-cell morphology (see below), whereas in other series, they are more uncommon.

Overall, our results affirm the primary trends in variant distribution established by other authors. This demonstrates the feasibility of reliably subtyping MIBC by immunohistochemical means, since the obtained IHC profiles largely recapitulate the transcriptomic classes. Some deviations could warrant further study—for example, the significantly lower share of stroma-rich tumors in our series, as well as the slightly higher percentages of neuroendocrine-like cases. These differences merit attention but do not alter the overall picture of concordance.

### 4.2. Correlation Between Histological Subtypes and Molecular Variants

Along with variant frequencies, the interrelationship between the morphological (histological) appearance of the tumor and its molecular profile is of particular interest. Increasing evidence shows that the different histological subtypes of urothelial carcinoma are not evenly distributed among molecular variants but rather exhibit preferences for certain profiles. In other words, morphologically distinct “divergent differentiations” (e.g., squamous, glandular) largely correlate with the variant determined by gene expression. Our observations confirm these patterns of association (Table 3). Below, we summarize the main histo-molecular correlations, comparing the literature data with the present results.

**Figure 3:**
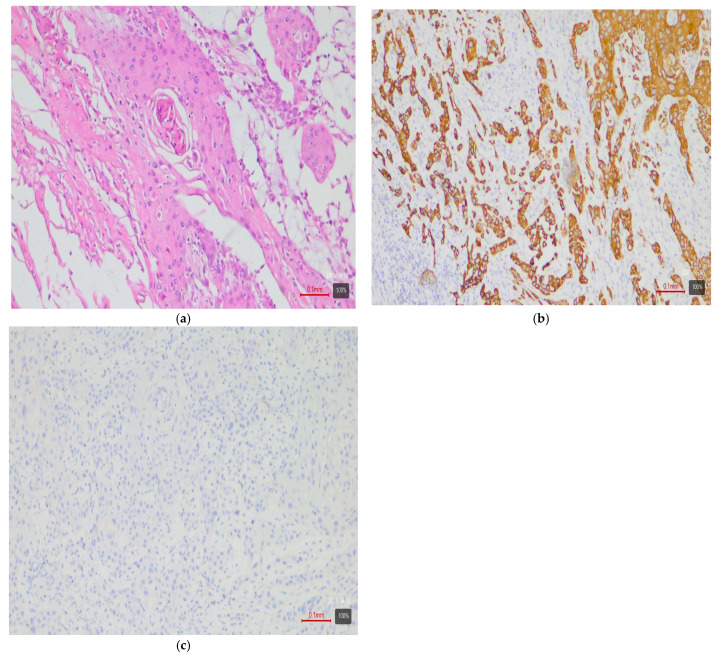
UC with squamous differentiation—basal variant. (**a**) H&E; (**b**) CK5/6 (+ve); (**c**) CK20 (−ve).

**Figure 4:**
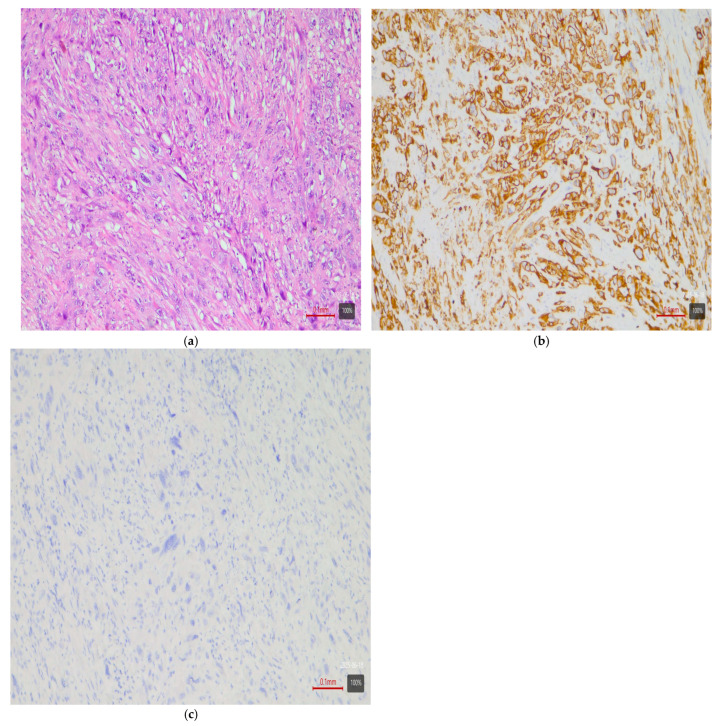
Sarcomatoid subtype—basal variant. (**a**) H&E; (**b**) CK5/6 (+ve); (**c**) CK20 (−ve).

**Figure 5:**
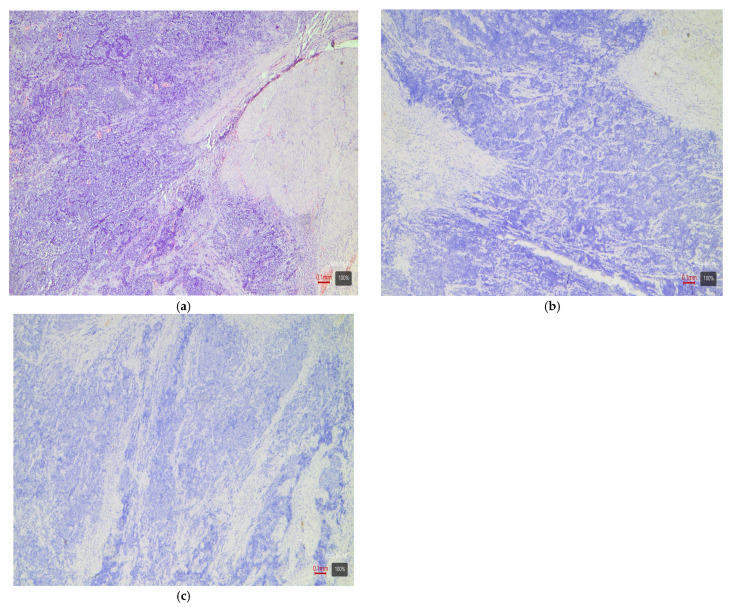
Small-cell carcinoma—neuroendocrine-like variant. (**a**) H&E; (**b**) CK5/6 (−ve); (**c**) CK20 (−ve).

**Figure 6:**
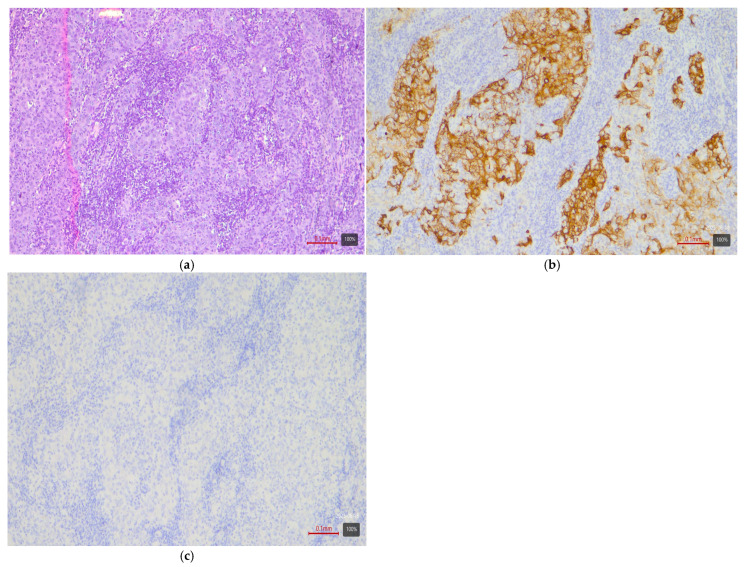
Lymphoepithelioma-like subtype—basal variant. (**a**) H&E; (**b**) CK5/6 (+ve); (**c**) CK20 (−ve).

- Papillary tumors—luminal variant. Urothelial carcinomas with papillary architecture (Figure 2) almost always belong to the luminal molecular class. It is well established in the literature that luminal bladder carcinomas often arise via a so-called “papillary pathway” (from papillary precursor lesions) and carry characteristic mutations in the FGFR3 gene [6]. In agreement with this, our data show that papillary urothelial carcinomas are entirely luminal in molecular profile. In 92% of cases, they fell into one of the luminal variants, most often luminal papillary (63% of papillary tumors)—Table 3. The rarely encountered nested subtype also follows this pattern—despite its “benign” morphological appearance, its expression profile is close to luminal tumors with frequent TERT mutations [6,9]. Both cases of nested carcinoma in our series are classified as luminal papillary variant (100%)—Table 3. These results confirm that conventional papillary carcinomas (including the nested subtype) belong almost exclusively to the luminal molecular group.
- Micropapillary subtype—luminal unstable variant. Micropapillary urothelial carcinoma is an aggressive histological subtype, but it too usually falls into the luminal spectrum of the molecular classification [4,5,7,9]. Tumors with micropapillary morphology exhibit the IHC profile of a luminal tumor (CK20-positive, CK5/6-negative) and are often classified transcriptomically as luminal (lumNS) variant [4,5,7,9]. In the consensus classification, the luminal non-specified variant (LumNS) is enriched specifically with micropapillary histology and is associated with a poorer prognosis. Our data correspond to these observations—75% of micropapillary tumors in the series are classified as luminal unstable variant, and the remaining 25% as luminal papillary. Not a single micropapillary tumor showed a basal or double-negative profile. This confirms the strong link of this histological subtype to the luminal molecular lineage.
- Squamous differentiation—basal variant. There is an exceptionally strong correlation between the presence of a squamous component in a tumor and the basal molecular phenotype (Figure 3). Basal MIBCs, by definition, have a gene signature overlapping with that of normal basal cells and often demonstrate squamous morphology [5]. Various studies note that “squamous differentiation is a common feature of basal tumors” [5]. Our investigation fully corroborates this trend: all 17 tumors with squamous differentiation are of the basal molecular variant (100%) (Table 3). This finding is in complete accordance with the literature and underscores that the presence of a substantial squamous component within a urothelial carcinoma virtually guarantees membership in the basal (basal/squamous) molecular class. Clinically, identification of a basal variant may be significant, since basal tumors are associated with more aggressive behavior and better response to chemotherapy.
- Glandular differentiation—luminal variant. Urothelial carcinoma with glandular differentiation is relatively uncommon. Due to the limited number of cases, the literature does not provide a definitive classification for this variant [5]. However, the available data suggest that in mixed tumors, the glandular component is more often encountered in a luminal context [4,5,7,9]. In other words, there is a tendency toward a luminal profile in cases with glandular differentiation. Our results support these facts—although based on only three cases, the observation is that two out of three tumors with a glandular component are classified as luminal unstable variant (the remaining one is basal). This points to a predominantly luminal orientation, though a basal profile may be present in a subset of these tumors. More cases are needed for firm conclusions, but at present, glandular differentiation is most frequently associated with a luminal molecular background.
- Sarcomatoid subtype—basal or stroma-rich variant. Sarcomatoid urothelial carcinoma is an extreme manifestation of tumor dedifferentiation, in which the epithelial tumor acquires a sarcomatous appearance. Molecularly, data indicate heterogeneity: a portion of sarcomatoid tumors represent an extreme manifestation of the basal variant (Figure 4) (with a tendency toward epithelial-mesenchymal transition, EMT), while others fall into a separate stroma-rich (“mesenchymal-like”) class. Choi et al. noted that basal tumors are enriched with sarcomatoid morphological characteristics [5]. Ravanini et al., via an IHC panel (CK5/CK20), showed that 88% of sarcomatoid MIBCs have either a basal (67%) or double-negative (33%) profile [5]. Consistently, our data demonstrate 60% basal and 40% stroma-rich sarcomatoid tumors, which essentially reproduces the literature values (Table 2). This confirms that the sarcomatoid subtype does not have a single molecular analog—most cases fall in the basal spectrum, but a significant portion have a distinct mesenchymal (non-basal, non-luminal) profile. Biologically, it is hypothesized that the sarcomatoid transformation may result from the progression of a basal tumor that has undergone EMT. Interestingly, sarcomatoid cases often harbor mutations in cell adhesion genes (e.g., CDH1) and loss of epithelial markers (such as EPCAM), accompanied by expression of mesenchymal factors (e.g., SNAI2)—an observation supporting the idea of a transition from a basal to a mesenchymal profile.
- Plasmacytoid subtype—luminal variant (with heterogeneity). The plasmacytoid urothelial carcinoma (also known as the diffuse subtype) is rare, composed of discohesive tumor cells resembling plasma cells. Molecularly, the majority of studies position it in the luminal spectrum. For example, Eckstein et al. found in plasmacytoid tumors an immunophenotype of CK20+/CK5/6– and GATA3+, which is characteristic of the luminal variant [5]. In a large series of 32 patients with the plasmacytoid subtype, a significantly lower level of basal markers CK5/6 and p63 was demonstrated compared to conventional urothelial carcinoma, alongside high expression of luminal markers GATA3 and CK20 [5]. Additionally, plasmacytoid tumors often exhibit HER2 amplification/overexpression, similar to other luminal carcinomas [5]. All of this supports the notion that the plasmacytoid subtype is predominantly luminal [5,7,9]. Our results generally concur with this—none of the three plasmacytoid tumors was basal or purely double-negative. In 100% of cases, the plasmacytoid tumors fell into luminal variants, albeit distributed among different subcategories (approximately one case in lumP, one case in lumNS, and one case in basoluminal variant)—see Table 3. No broad generalizations can be made due to the small number, but the observed variability suggests that while most plasmacytoid tumors are luminal, individual cases can fall into intermediate categories (including mixed profiles). There is evidence that small series have identified individual plasmacytoid tumors with co-expression of basal markers as well—e.g., classified as genomically unstable (GU) or Lund’s Uro B variant [5]. In our cohort, the three cases illustrate precisely this heterogeneity. Nevertheless, the overall pattern—a predominantly luminal phenotype—holds, and not a single plasmacytoid carcinoma showed a purely basal or double-negative profile.
- Small-cell carcinoma—neuroendocrine-like variant. Small-cell carcinoma of the bladder is a distinct, rare histological subtype with neuroendocrine morphology. Molecularly, it corresponds to the aforementioned “neuronal” (neuroendocrine) variant, identified by TCGA as the smallest group [11]. It is characterized by frequent mutations in the tumor suppressors TP53 and RB1. In the consensus classification, these tumors fall into the neuroendocrine-like (Ne-like) class. As expected, our data show full concordance: all five cases of small-cell carcinoma and one poorly differentiated urothelial with small-cell morphology in this study are classified as double-negative variant with neuroendocrine morphology (100%)—i.e., these tumors do not express luminal or basal differentiation markers but have a distinct transcriptional signature (Figure 5). The correlation here is extremely strong, since even at the histological level, small-cell carcinomas are recognized as different from urothelial carcinoma; the molecular classification confirms this by segregating them into a separate category (“neuronal”).
- Rare variants. Some rarer histological variants of urothelial carcinoma are encountered too sporadically to make a compelling correlation, but available evidence also suggests preferred profiles. For example, the lymphoepithelioma-like subtype (urothelial carcinoma resembling lymphoepithelioma in the nasopharynx) is more associated with a basal/immune-infiltrated phenotype [5]. In our cohort, we had one case of a mixed urothelial carcinoma including a lymphoepithelioma-like component, which showed a basal immunophenotype (Figure 6). The microcystic subtype (characterized by formation of small cystic spaces) may have mixed features—some authors assign it to the luminal class, while others assign it to the basal class [11]. In our samples, we had two cases of a microcystic subtype as part of a mixed tumor; one demonstrated a luminal unstable and the other a basal immunophenotype. In our series, these rare variants are observed only as parts of mixed tumors; they are few in number, and no independent conclusions can be drawn.
- Mixed subtypes (with basoluminal profile). A significant proportion of cases—27%—have a heterogeneous morphology containing more than one of the above-mentioned histological components. In these cases, a mixed immunophenotype with co-expression of basal and luminal markers is often observed. In fact, our defined basoluminal category (9% of all tumors) reflects exactly such cases with a “double positive” profile. Similar mixed expression profiles have been described in the literature—e.g., the Lund UroB variant, or other authors who note ~5–10% of tumors with a combination of basal and luminal characteristics. Our data (9%) are in line with these observations. Mixed basoluminal tumors often correspond morphologically to mixed histological subtypes. Such tumors underscore the internal heterogeneity of some MIBCs and demonstrate that molecular variants are not always strictly demarcated—overlap within the same tumor is possible. From a practical standpoint, in these cases, the morphological and IHC assessment should account for all components in order to characterize the complete profile of the neoplasm.

## 5. Conclusions

The correlations outlined above indicate that the morphological subtypes of urothelial carcinoma largely predetermine the molecular variant. The overall picture demonstrates more concordance than discordance: papillary tumors correspond to the luminal axis and tumors with squamous differentiation to the basal axis of the molecular classification. Deviations from these main “axes”—such as mesenchymal (stroma-rich, sarcomatoid) or neuroendocrine morphology—represent separate variants that can also be recognized and predicted by an experienced pathologist. This knowledge has practical significance. For example, the presence of a squamous-differentiated tumor suggests a basal type, for which we would consider more aggressive systemic therapy, whereas a purely papillary tumor is most likely luminal and is characterized by a better prognosis but also a weaker response to chemotherapy. Thus, the combination of classic histopathology with molecular (IHC) profiling provides the most complete characterization of the tumor and may help achieve a more precise prognosis and personalized treatment for patients with muscle-invasive bladder cancer.

## Figures and Tables

**Table 1:** Frequency of the main histological subtypes in the studied cohort (*n* = 100). NOS: not otherwise specified (high-grade carcinoma without special features).

Subtype (WHO 2022)	Number of Cases (*n*)	Percentage (%)
UC (NOS)	4	4.0%
Papillary UC	27	27.0%
UC with squamous diff.	17	17.0%
UC with glandular diff.	3	3.0%
Sarcomatoid	5	5.0%
Plasmacytoid	3	3.0%
Nested	2	2.0%
Micropapillary	4	4.0%
Small-cell carcinoma	5	5.0%
Poorly differentiated UC	3	3.0%
Mixed tumor (≥2 subtype)	27	27.0%
Total	100	100%

**Table 2:** Comparison of the frequency of MIBC molecular variants between the present study (N = 100) and published data (consensus classification 2020 and The Cancer Genome Atlas, TCGA 2017).

Molecular Variant	Our Study (N = 100)	Number of Cases (*n*)	Consensus 2020 (N ≈ 1750)	TCGA 2017 (N = 408)
Ba/Sq	33%	33	35%	35% (Ba/Sq)
LumPap	24%	24	24%	35% (LumPap)
LumU	16%	16	15%	Inc. in Lum-infiltrated
LumNS	10%	10	8%	6% (Luminal)
Stroma-rich	2%	2	15%	19% (Lum-infiltrated)
NE-like	6%	6	3%	5% (Neuronal)
BasoLum	9%	9	No separate class	No separate class

**Table 3:** Comparison of the predominant molecular variants in various histological subtypes of urothelial carcinoma (literature data) and their distribution in our study (N = 100). *Notes:* (1) luminal = includes all luminal variants (papillary, unstable, non-specified); (2) double-negative = absence of expression of typical luminal or basal markers.

Histological Subtype	Dominant Molecular Variant Literature [4,7,9]	Most Common Variant (Our Data)
Papillary urothelial carcinoma	Luminal (especially LumP)	LumP 63% of cases (17 cases from 27)
Micropapillary	Luminal (e.g., LumNS or LumU)	LumU 75% of cases (3 cases from 4)
Nested subtype	Luminal or Basal	LumP 100% of cases (2 cases from 2)
UC with squamous diff.	Basal (Ba/Sq)	Basal 100% of cases (17 cases from 17)
UC with glandular diff.	Luminal (limited data; likely lumU)	LumU 67% of cases (2 cases from 3)
Sarcomatoid	Basal	Basal (60%; 3 cases)), Stroma-rich (40%; 2 cases) total 5 cases
Plasmacytoid	Luminal (predominantly lumNS) Stroma rich	No dominant; ~evenly distributed among 1 LumP, 1 basolum., and 1 lumNS (~33% each/total 3 cases)
Small-cell carcinoma	NE-like (“neuronal”)	NE-like (100%; 5 cases from 5)

## Data Availability

The original contributions presented in this study are included in the article. Further inquiries can be directed to the corresponding author.

## Abbreviations

The following abbreviations are used in this manuscript:

BC	Bladder carcinoma
MIBC	Muscle-invasive bladder carcinoma
TCGA	The Cancer Genome Atlas
IHC	Immunohistochemistry
TURB	Transurethral resection
RC	Radical cystestomy
FGFR	Fibroblast Growth Factor Receptor 3
